# Screening for Gestational Diabetes during the COVID-19 Pandemic—Current Recommendations and Their Consequences

**DOI:** 10.3390/medicina57040381

**Published:** 2021-04-15

**Authors:** Anca Maria Panaitescu, Anca Marina Ciobanu, Maria Popa, Irina Duta, Nicolae Gica, Gheorghe Peltecu, Alina Veduta

**Affiliations:** 1 Carol Davila University of Medicine and Pharmacy, 020021 Bucharest, Romania; ciobanu.ancamarina@gmail.com (A.M.C.); gica.nicolae@umfcd.ro (N.G.); gheorghe.peltecu@umfcd.ro (G.P.); 2 Filantropia Clinical Hospital, 11171 Bucharest, Romania; mariapopa.og@gmail.com (M.P.); alina.veduta@gmail.com (A.V.); 3 Endocrinology and Diabetes, Southend University Hospital, Essex SS0 0RYUK, UK; irinaduta83@yahoo.com

**Keywords:** gestational diabetes mellitus, guidelines, risk factors, screening, impact, COVID-19

## Abstract

Gestational diabetes mellitus (GDM) is recognized as one of the most common medical complications of pregnancy that can lead to significant short-term and long-term risks for the mother and the fetus if not detected early and treated appropriately. Current evidence suggests that, with the use of appropriate screening programs for GDM, those women diagnosed and treated have reduced perinatal morbidity. It has been implied that, when screening for GDM, there should be uniformity in the testing used and in further management. This paper summarizes and compares current screening strategies proposed by international bodies and discusses application in the context of the COVID-19 pandemic.

## 1. Introduction

Gestational diabetes mellitus (GDM) is recognized by the American College of Obstetricians and Gynecologists (ACOG) to be one of the most common medical complications of pregnancy [1] that can lead to significant short-term and long-term risks for the mother and the fetus [2], if not detected early and treated appropriately. Many studies and guidelines have been focused on the matter of GDM and it is generally agreed that it represents an important public health issue due to its significant clinical and economic burden [3].

It has been observed that the incidence and the prevalence of GDM are increasing directly proportional with the increase in the prevalence of obesity and sedentary lifestyle in women at child-bearing age [1,4]. The number of metabolic risk factors present in pregnant women directly influences the incidence of GDM [5]. Moreover, GDM and obesity are both independently associated with maternal and fetal complications, but their combination has an even greater impact on pregnancy outcomes [6]. However, studies and guidelines outline the importance of early recognition of GDM and suggest that associated maternal and fetal complications could be reduced and even prevented through timely intervention—dietary measures, drug therapy and appropriate fetal monitoring [7].

In 2019, the International Diabetes Federation estimated that one in six live births are affected by hyperglycemia in pregnancy, with GDM accounting for the majority of these cases (17 million globally per year) [8].

Despite the fact that there is extensive research on GDM and glucose metabolism disorders in pregnancy, most aspects of the matter remain controversial. A variety of algorithms for the screening, diagnosis, management and follow-up are advocated by different health organizations. These variations generate major differences in the reported prevalence, complications, efficacy of treatment and follow-up of GDM [4].

Our purpose is to review the recent studies and the current international guidelines concerning GDM screening and to discuss the consequences of their recommendations, including the temporary guidelines released during the COVID pandemic and their impact on the detection rate or pregnancy outcome.

## 2. Definition of Gestational Diabetes

Traditionally, the American Diabetes Association (ADA) [9] defined GDM as “any degree of glucose intolerance first recognized during pregnancy, regardless of the magnitude of hyperglycemia”. However, later studies have led ADA to affirm that it is the severity of hyperglycemia that counts when defining GDM, assuming that short-term and long-term maternal and fetal risks depend on it [9].

Currently, hyperglycemia detected during pregnancy is segregated into either diabetes in pregnancy or GDM [10,11]. Diabetes in pregnancy applies to pregnant women who have preexisting diabetes or overt diabetes—first noted during pregnancy—that meet the World Health Organization (WHO) standard criteria (for nonpregnant state) [11].

Gestational diabetes is defined as hyperglycemia recognized most likely after 24 weeks of pregnancy [12]. Unlike overt diabetes, GDM usually is a transient hyperglycemic state associated with pregnancy that usually resolves once the pregnancy ends [4], but it tends to reoccur in subsequent pregnancies.

## 3. Current Available Screening and Diagnosis Methods

Generally, it is challenging to identify and accurately distinguish GDM from preexisting diabetes, mainly because the majority of women are not screened for diabetes preconceptionally [1]. Yet, the Australasian Diabetes in Pregnancy Society (ADIPS) [13] points out that women affected by glucose metabolism disorders during the gestational period, whether symptomatic or not, necessitate immediate attention, this implying evaluation for potential complications of undiagnosed diabetes, such as retinopathy and nephropathy in mothers, and congenital defects in fetuses [4,14]. However, the appropriate moment and method of screening is still a subject of dispute [4].

### 3.1. First Trimester Screening

Several guidelines suggest that first prenatal care visit is the right opportunity to assess the risk factors for diabetes [1,9,15]. Early universal testing or limiting testing to those pregnant women classified as high risk (based on the risk factors identified) is still under debate [14]. The majority of guidelines recommend screening for **overt diabetes**, particularly in overweight or obese women with or without additional diabetic risk factors [1,9,10,13,15].

From another point of view, it was suggested that early screening should depend on the prevalence of type 2 diabetes. As a consequence, in populations with a high prevalence of type 2 diabetes, universal early testing is recommended [14]. Nevertheless, there is evidence that the prevalence of diabetes in reproductive-age women differs considerably in countries around the world.

In Romania, as well as in other European countries, USA, Canada and Australia, early screening is only performed in the presence of risk factors. The current Romanian guideline recommends the assessment of diabetes risk factors during the first prenatal consultation. Pregnant women at high risk are screened for overt type 2 diabetes without delay after confirmation of pregnancy [15] (the criteria for high risk of diabetes are noted in Table 1). Testing is not required in pregnant women at low risk of developing GDM. In Romania, pregnant women are mainly young, having one of the highest adolescent birth rates in Europe, of white race, with low frequency of obesity at the beginning of pregnancy and relatively low incidence of hypertensive disorders associated to pregnancy, therefore it can be considered a low-risk population for gestational diabetes [16,17].

The International Association of the Diabetes and Pregnancy Study Groups (IADPSG) has made recommendations regarding the diagnosis of overt diabetes. The diagnosis is established by identifying one or more of the following criteria: fasting plasma glucose (FPG) ≥126 mg/dL (≥7 mmol/L), HbA1c ≥ 6.5% (≥48 mmol/mol) or random plasma glucose ≥ 200 mg/dL (≥11.1 mmol/L), which needs confirmation by FPG or HbA1c [14]. However, the World Health Organization (WHO) [10] argues that **HbA1c** is not accurate for the diagnosis of diabetes in pregnancy. Furthermore, it suggests that **FPG** values in early pregnancy ≥92 mg/dL (5.1 mmol/L) should be classified as **GDM** [10].

#### Risk Factors

Selective screening of diabetes is recommended by several guidelines—National Institute for Health and Care Excellence (NICE) [18], Scottish Intercollegiate Guidelines Network (SIGN) [19], Romanian guideline [15] and the National College of French Obstetrician Gynecologists and French-Speaking Diabetes Society (CNGOF and SFD) [20]. It should be based on the risk factors identified at the first prenatal visit (Table 1). Yet, there is no consensus on which of these risk factors are the most reliable in detecting GDM. Moreover, some of the risk factors proposed have different definitions in different guidelines [3] (Table 1).

**Table 1:** Risk factors for diabetes proposed by different guidelines.

Committee of the Romanian Ministry of Health for Diabetes, Nutrition and Metabolic Diseases [15]	ADA [9]
***High* risk factors for GDM** Severe obesity Previous GDM or previous macrosomic baby Persistent glycosuria PCOS Significant family history of type 2 diabetes ***Low* risk factors for GDM** Maternal age <25 Normal weight before pregnancy Member of an ethnic group with a low risk of gestational diabetes No family history of diabetes No personal history of glucose intolerance	BMI > 25 kg/m^2^ Previous history of GDM Family history of diabetes (1st degree relative) Previous macrosomic child >9 lb (4 kg) No physical activity Hypertension HDLc < 35 mg/dL (0.90 mmol/L) and/or triglyceride > 250 mg/dL (2.82 mmol/L) PCOS HbA1c ≥ 5.7% and previous IGT or IFG Signs of insulin resistance such as acanthosis nigricans History of cardiovascular disease Ethnic backgrounds: African American, Latino, Native American, Asian American, Pacific Islander
**ADIPS [13]**	**NICE and SIGN [18,19]**
***Moderate* risk factors for GDM** BMI 25–35 kg/m^2^ Ethnic backgrounds: Asian, Indian, aboriginal, Torres Strait Islander, Pacific Islander, Maori, Middle Eastern and non-white African ***High* risk factors for GDM** BMI > 35 kg/m^2^ Age ≥ 40 years Previous history of GDM Family history of diabetes History of high blood glucose History of macrosomic child ≥ 4.5 kg PCOS Medication: Corticosteroids, antipsychotics	BMI > 30 kg/m^2^ Previous GDM Family history of diabetes (first-degree family member) Previous macrosomic baby ≥ 4.5 kg Ethnic backgrounds South Asian (India, Pakistan or Bangladesh), Black Caribbean, Middle Eastern (Saudi Arabia, United Arab Emirates, Iraq, Jordan, Syria, Oman, Qatar, Kuwait, Lebanon or Egypt)
	**CNGOF and SFD [20,21]**
	BMI ≥ 25 kg/m^2^, maternal age ≥ 35years Personal history of GDM Family history of diabetes Previous macrosomic baby
**Canadian Diabetes Association [22]**	**FIGO [11]**
Age > 35 years High-risk ethnic origin: South Asian, African, Arab, Hispanic Corticosteroid medication BMI > 30 kg/m^2^ GDM in previous pregnancy Previous macrosomic baby ≥ 4.0 kg First-degree family history of diabetes PCOS or acanthosis nigricans	Older age, higher parity, ethnicity Obesity or excessive weight gain during current pregnancy History of GDM or macrosomia Family history of diabetes Previous poor pregnancy outcome Multiple pregnancy

CNGOF and SFD, National College of French Obstetrician Gynecologists and French-Speaking Diabetes Society; ADA, American Diabetes Association; ADIPS, Australasian Diabetes in Pregnancy Society; NICE and SIGN, National Institute for Health and Care Excellence and Scottish Intercollegiate Guidelines Network; BMI, body mass index; HDLc, high-density lipoprotein cholesterol; PCOS, polycystic ovary syndrome; HbA1c, glycated hemoglobin; IGT, impaired glucose tolerance; IFG, impaired fasting glucose, FIGO, International Federation of Gynecology and Obstetrics.

### 3.2. 24–28 Weeks Screening

#### 3.2.1. Universal Versus Selective Screening

There are many recent studies comparing universal and selective screening, the majority of them being in favor of universal screening [23,24,25,26].

Selective screening at 24–28 weeks’ gestation, based on the risk factors identified in the first trimester, is in use in many European countries, including Romania.

Farrar et al. [24] conducted a systematic review and meta-analysis of studies evaluating the risk factors for GDM, aimed to assess their predictive accuracy in identifying women predisposed to GDM. They concluded that risk factor-based screening has a weak potential to accurately identify those women. Moreover, they suggest that using this strategy will reduce the expected effect of antenatal GDM screening, testing and management programs [24]. Furthermore, a large cohort study [25] of 18,775 women, conducted in France, aimed to evaluate the selective risk factors-based screening strategy for GDM, concluded that this method would miss one third of the women with GDM [25]. Another study from Italy [26] suggests that the selective screening strategy should be used only in those situations when universal screening is not possible. It also outlines the importance of diagnosis and proper management of GDM in reducing the maternal and fetal morbidity and indicates that, even though the diagnosis and intensive treatment of GDM may cause higher initial costs for healthcare systems, during the long-term there are substantial financial savings [26].

Universal screening at 24–28 weeks of gestation, which is carried out in the USA and in some European countries, regardless of the risk factors, initially implies higher costs [16]. However, when compared to the selective screening strategy, it seems to be a better option, since the selective screening was proven to fail detecting an important number of cases of GDM [25]. Moreover, the universal screening was demonstrated to be cost-effective on the long-term, by avoiding the adverse outcomes of GDM pregnancies [26].

It has been suggested it would be worth considering universal screening for GDM at 24–28 weeks of gestation in Romania also, since the reported prevalence of type 2 DM is high [16,27].

#### 3.2.2. Methods

Currently, there are two strategies to diagnose GDM:

1. The **“one-step” approach** with 75 g oral glucose-tolerance (OGTT);
2. The **“two-step” approach** with 50 g nonfasting glucose screen, followed by 100 g OGTT for the patients who screen positive.

##### The HAPO Study and IADPSG Criteria

The **“one-step”** approach originates from the IADPSG recommendations, based on the HAPO study. The Hyperglycaemia and Adverse Pregnancy Outcomes (HAPO) [28] is the landmark observational study that led to recommendations for the criteria on the diagnosis of GDM. It was aimed at clarifying the risks of adverse outcomes associated with various degrees of maternal glucose intolerance [14,28]. It included a total of 25,505 pregnant women from a heterogeneous, multinational, multicultural, ethnically diverse population from 15 centers, in nine countries. Participants underwent a standard oral glucose-tolerance test, with the use of a 75-g dose of glucose, between 24 and 32 weeks of gestation (target time of testing: 28 weeks). Primary outcomes included: 1. birth weight above the 90th percentile for gestational age; 2. primary cesarean delivery; 3. clinical neonatal hypoglycemia; 4. cord-blood serum C-peptide level above the 90th percentile—fetal hyperinsulinemia. Secondary outcomes included: 1. preeclampsia; 2. premature delivery; 3. shoulder dystocia and birth injury; 4. need for intensive neonatal care; 5. hyperbilirubinemia.

The HAPO study demonstrated a continuous and graded relationship between maternal hyperglycemia and the increasing frequency of the primary outcomes, independent of other risk factors [28].

Based on the results of the HAPO study, the IADPSG [6] released a consensus statement in 2010, on new criteria for the diagnosis of GDM. The consensus stated: 1. the ”one-step” approach with 2 h, 75 g oral OGTT after overnight fasting of 8–14 h [10] should be used between 24–28 weeks of pregnancy; 2. one abnormal value is sufficient to diagnose GDM (cutoffs—fasting value, 92 mg/dL; 1 h value, 180 mg/dL; or 2 h value, 153 mg/dL).

In European countries and the USA, there are discrepancies with respect to the recognition and implementation of the IADPSG criteria and recommendations. The use of the IADPSG criteria is supported by the American Diabetes Association (ADA) since 2011, by the Endocrine Society and the World Health Organization (WHO) since 2013, and by the International Federation of Obstetrics and Gynecology (FIGO) since 2015 [29]. However, the American College of Obstetricians and Gynecologists (ACOG) and the National Institute of Health (NIH) promote the older **“two-step”** approach screening strategy, using the nonfasting 50 g glucose challenge test. An abnormal test implies a plasma glucose measured at 1 h after the load ≥130 mg/dL (7.2 mmol/L), 135 mg/dL (7.5 mmol/L) or 140 mg/dL (7.8 mmol/L). If the 50 g glucose challenge test is positive, it should be followed by the 3 h, 100 g OGTT using the Carpenter and Coustan diagnostic criteria [30], performed when the patient is fasting. Gestational diabetes is diagnosed when at least two of the following criteria are met: fasting ≥95 mg/dL (5.3 mmol/L), 1 h value ≥ 180 mg/dL (10.0 mmol/L), 2 h value ≥155 mg/dL (8.6 mmol/L), 3 h value ≥140 mg/dL (7.8 mmol/L) [30].

The Canadian Diabetes proposes two approaches for screening. The preferred one consists of two steps, the 50 g glucose challenge test with plasma glucose level measured within 1 h, followed by OGTT with 75 g glucose if the first glucose level is 140–199 mg/dL (7.8–11.0 mmol/L). The criteria for GDM are FPG ≥95 mg/dL (5.3 mmol/L), 1 h value ≥ 190 mg/dL (10.6 mmol/L), 2 h value ≥162 mg/dL (9 mmol/L). The alternative approach is to perform the OGTT with 75 g glucose directly [22,31].

Table 2 summarizes the various criteria that are currently in use for the diagnosis of GDM (Table 2). The reader should be aware that other criteria for the diagnosis of GDM have been used over time, which represents a significant challenge for the systematic reviews and meta-analyses on the subject [32].

Currently, there is an intense dispute on the fact that WHO [10], FIGO [11], IADPSG [14] and ADA [9], recommend the “one-step” approach, while ACOG [1] and NIH [31] recommend the “two-step” approach. It was hypothesized that the “one-step” approach would considerably raise the incidence of GDM [32] and this fact could lead to an additional burden on the healthcare systems [33]. This hypothesis led Saccone et al. to conduct a systematic review and meta-analysis [32] comparing the two approaches. They assessed the incidence of maternal and neonatal adverse outcomes and found that the diagnosis of GDM using the “one-step” approach results in better perinatal outcomes (lower incidence of LGA, NICU admission, neonatal hypoglycemia) when compared to the “two-step” approach [32]. However, they noticed no differences in the incidence of GDM. Another study aimed to evaluate specifically the incidence of GDM using the “one-step” in comparison with the “two-step” method also found similar incidence of GDM, using the two methods in parallel [33].

With increasing maternal age and obesity, there is an increase in the prevalence of gestational diabetes, with variable rates from less than 5% in East Asian population to greater than 45% is South Asian population [34]. Moreover, adopting new IADPSG/WHO criteria resulted in more women diagnosed with gestational diabetes. The increase of positivity test is significantly related to ethnicity—women of Asian and Middle Eastern background being at higher risk than white European women. New evidence suggests that the criteria recommended by UK NICE guideline might underestimate the prevalence of gestational diabetes, especially in South Asian women [35], therefore, for this group of patients, lower thresholds of fasting and postload glucose are proposed for diagnosing gestational diabetes [36]. Despite cost effectiveness concerns, increasing the detection rate would identify more pregnancies at increased risk of adverse perinatal outcome and adequate follow-up, lifestyle changes and safe medications are effective not only in avoiding short-term adverse outcomes, but also in improving the long-term maternal and offspring complications associated to gestational diabetes.

## 4. The Importance of Screening for GDM and the Impact on Outcomes

Questions and doubts were expressed regarding the cost-effectiveness of different types of screening methods and their consequences. Current evidence suggests that, with the use of appropriate screening programs for GDM, those women diagnosed and treated have reduced perinatal morbidity. It has been implied that, when screening for GDM there should be uniformity in the testing used and in the further management [37]. Screening and quickly identifying women with GDM are key elements of an early prevention strategy, not only for better neonatal outcomes, but also for better short-term and long-term outcomes for the mother.

If we consider the *short-term* consequences (Table 3), women with GDM are known to have a greater risk of adverse pregnancy outcomes. Treatment of GDM diminishes the frequency of macrosomia, prematurity, the risk of preeclampsia and C-section delivery. Moreover, treatment of GDM is associated with a significant decrease in the rate of important newborn complication, such as perinatal death, NICU admission, neonatal hypoglycemia, shoulder dystocia and birth trauma, including fracture or nerve palsy [1,38]. Accordingly, if we analyze the cost-effectiveness of screening, diagnosing and treating GDM, a reduction in short-term morbidities may result already in a decrease in the costs of care for women with GDM [39]. Women diagnosed with gestational diabetes by the IADPSG criteria but not by other less strict criteria are at increased risk for preeclampsia and macrosomia, and for cesarean delivery and preterm birth [40]. There are no direct treatment studies on this specific group of patients, but the “one step” (IADPSG) approach, in general, has been associated with better maternal and perinatal outcomes compared with the “two step” approach, in randomized studies [40,41,42,43,44].

In the *long-term* (Table 3.), if we were to look at *mother* outcomes, the HAPO follow-up study [45] has shown that GDM carries a major risk of developing this condition in subsequent pregnancies, with a frequency of 14.3%. Moreover, after 10–14 years of follow-up, women with GDM have a significant higher risk of developing type 2 diabetes and prediabetes, independent of other variables [45]. Women with gestational diabetes are at increased risk of developing type 2 diabetes later in life [46,47,48,49,50,51], risk which was reported to be 20 times higher than in women without gestational diabetes, depending on the patient characteristics such as age, race, weight or family history of diabetes. These women present some particular changes in glucose regulation due to resistance to insulin effects on glucose clearance and production. There is a 6% higher glucose production and 9% lower glucose clearance after an overnight fast compared with normal pregnant women. Moreover, women with GDM had a 67% impairment of pancreatic beta-cell compensation for insulin resistance compared with normal pregnant women [52]. Pregnancy-related factors are also significant predictors of progression to type 2 diabetes, such as insulin requirement and elevated HbA1c [53].

Two recent meta-analyses (Kramer et al. 2019 and Li et al. 2018) showed a two-fold increase in the risk for cardiovascular disease for women with prior GDM, when compared to women without GDM [54,55].

Strictly concerning the cost-effectiveness of screening strategies, it has been hypothesized that limiting diagnostic testing only to those women presenting risk factors for GDM, may be, initially, less expensive than testing all pregnant women [24]. On the other hand, if all pregnant women undergo the OGTT, there is a better chance to identify more cases of GDM, and consequently to reduce the adverse outcomes by treating them [24]. Promptly applying the intervention strategies for these patients reduce the long-term risks of not only type 2 diabetes, but also the other associated complications such as cardiovascular disease [56].

In the *long-term*, if we were to look at *offspring* outcomes, the HAPO follow-up study (evaluating the glucose status of 4160 children of GDM mothers) [45] has shown that previous GDM diagnosis is associated with increased risk of childhood overweight and obesity. In terms of children body composition, hyperglycemia during pregnancy was associated with statistically significant increase of body fat in offspring. Moreover, if exposed to untreated GDM, the offspring were at high risk of developing insulin resistance, the results remaining significant even after adjusting the children’s body mass index. Data from HAPO follow-up confirm older observations on the effect of maternal GDM on children development and health [57]. Epigenetics now provides explanations for in utero developmental programming [58,59]. Efficient management of GDM has been shown to significantly improve the fasting glucose and insulin resistance in female offspring at 5–10 years of follow-up [60].

About 16% of pregnancies are affected by GDM and 50% of these women do not have any risk factors. Considering the significant adverse effects of GDM, early diagnosis is particularly important in order to minimize the maternal and fetal adverse outcomes, by acting promptly through dietary measures, drug therapy and appropriate fetal monitoring [7].

## 5. Coronavirus Pandemic and Antenatal Screening for GDM

In the actual pandemic context with SARS-CoV-2 infection, in an attempt to reduce the risk of contamination in pregnant women and to restrict the contact with a highly infectious environment such as hospitals, international authorities have recently released new guidelines and recommendations regarding some medical services now considered unessential in the antenatal care. Some of those recommendations refer to screening for gestational diabetes. Although GDM is one of the most common medical complications of pregnancy, leading to a higher rate of neonatal and maternal adverse outcomes when undiagnosed and inadequately controlled, new strategies of screening for GDM have been proposed (Table 4), strategies with a significantly lower detection rate [62,63]. Canadian revised pandemic guidelines accept HbA1c > 5.7% (39 mmol/mol) and/or RPG > 200 mg/dL (11.1 mmol/L) as GDM [64]. Amended Australian guidelines recommend FPG and progression to OGTT only for levels between 85 and 90 mg/dL (4.7–5.0 mmol/mol), while a FPG > 92 mg/dL (5.1 mmol/mol) confirms definitive diagnosis of GDM [65]. These strategies are missing an important number of GDM cases [62,63,64,65] which probably will reflect in poorer outcomes for mothers and babies. Healthcare systems are actively considering the risks involved by not screening for GDM. For example, in May 2020, a RCOG guideline in the context of COVID pandemic temporarily replaced the traditional OGTT test and the new recommendation stated that women considered being at high risk according to NICE criteria should be tested at booking by HbA1c and RPG and if the results are borderline, HbA1c 41–47 mmol/L or RPG 9–11 mmol/L, the patients should be considered as having GDM. Further, women at high risk should undertake at 28 weeks HbA1c and either FPG or RPG. The new criteria for diagnosis of GDM were HbA1c > 5.7% (39 mmol/mol) or a FPG > 95 mg/dL (5.3 mmol/L) or RPG > 162 mg/dL (9 mmol/L).

Following implementation of new recommendations, subsequent observational studies reported the detection rate of such an approach. The data indicate that the proportion of women diagnosed with GDM dropped from 7.7% to 4.2%, and the new screening method failed to detect 57% of cases. When negative women were reassessed by performing the standard 2 h, 75 mg glucose tolerance test, 20% of them were diagnosed with gestational diabetes. Therefore, the evidence does not support the general implementation of new screening method outside the COVID pandemic period [66]. The same trend of reduced frequency of GDM was reported when using new pandemic screening algorithm proposed by Canadian (82% reduction to 2.5%) and Australian (25% to 12.7%) guidelines. Despite a lower frequency of GDM based on new criteria, a secondary analysis of HAPO data from five HAPO centers reported that adverse outcomes related to GDM, including LGA neonates or operative delivery, are less common in those women diagnosed as having GDM based on elevated 1 or 2 h OGTT glucose, but with a fasting glucose <4.7 mmol/L [67]. This observation suggests that the temporary screening algorithm might not have a great impact on the pregnancy outcomes, but it aims to avoid frequent prenatal visits and possible exposure to SARS CoV-2 virus, which might have a greater impact on maternal prognosis, delivery and the health care system. However, any new screening methods should be tested on prospective randomized studies before generally implementing in clinical practice.

This recommendation was gradually modified toward the traditional system of screening for GDM. Australia, facing fewer COVID-19 cases than the UK or Canada, have already modified the screening recommendations, returning to routine OGTTs where COVID-19 transmission risk is considered low. In December 2020, a new RCOG guideline clarified that OGTT should be the default screening method for gestational diabetes in pregnancy, unless it is unsafe to offer it [68,69].

Postpartum management has also been adapted to the new pandemic restrictions; therefore postpartum screening for type 2 DM in women with GDM has been delayed by all of the available national guidelines during the pandemic. Postpartum screening is postponed to 3–6 months after delivery using HbA1c only according to the UK guideline, while the Australian guideline recommended delaying the postpartum OGTT for 6–12 months [65,68].

## 6. Conclusions

Currently, there is still much controversy surrounding both the screening and the diagnosis of GDM.

Uniformity in diagnostic criteria and screening strategies for GDM is desirable to align preventive and therapeutic strategies.

The implementation of an appropriate strategy of screening, treatment and follow-up for women with GDM is of utmost importance in reducing the risk of associated complications.

The “one-step” approach is the preferred method for the diagnosis of GDM, being recommended by the majority of the guidelines. Universal screening of GDM in all pregnant women is desirable in Romania and worldwide. Even though it may have higher costs initially, evidence suggests that it is cost-effective in the long-term because intervention strategies could prevent the occurrence of type 2 diabetes and the associated cardiovascular complications, thus leading to the improvement of the health of the general population.

Regarding the newly proposed screening methods during the COVID- 19 pandemic, we are aware of the increased risk of viral spread in the maternity units, but we believe that missing the OGTT could have detrimental consequences with GDM being overlooked at a cost of maternal, fetal and neonatal complications. Vaccination of healthcare workers and pregnant women, adapting antenatal clinic logistics and maintaining public health measures would allow rapid return to adequate screening for GDM, resuming proper follow-up interventions, not missing the valuable opportunity to detect those that are at high risk for developing type 2 diabetes in later life and to offer postpartum interventions.

## Figures and Tables

**Table 2:** Diagnostic criteria for GDM proposed by different guidelines [31].

Approach	Criteria	Fasting mg/dL	1 h mg/dL	2 h mg/dL	3 h mg/dL
Two step (100 g load)	Carpenter and Coustan	95 (5.3 mmol/L)	180 (10.0 mmol/L)	155 (8.6 mmol/L)	140 (7.8 mmol/L)
Two step (75 g load)	CDA	95 (5.3 mmol/L)	191 (10.6 mmol/L)	160 (8.9 mmol/L)	
One step (75 g load)	WHO	92 to 125 (5.1 to 6.9 mmol/L)	180 (10.0 mmol/L)	153 to 199 (8.5 to 11 mmol/L)	
	IADPSG	92 to 125 (5.1 to 6.9 mmol/L)	180 (10.0 mmol/L)	153 (8.5 mmol/L)	
	NICE	100 (≥5.6 mmol/L)		140 (≥7.8 mmol/L)	

CDA: Canadian Diabetes Association; WHO: World Health Organization; IADPSG: International Association of Diabetes and Pregnancy Study Groups.

**Table 3:** Maternal and offspring short- and long-term complications after gestational diabetes [61].

	Maternal	Offspring
**Short-term complications**	Gestational hypertension	Macrosomia
	Preeclampsia	Shoulder dystocia, birth trauma
	Cesarean delivery	Hypoglicemia
	Perineal injury	Prematurity
		Fetal distress, adverse perinatal outcome, NICU admission
		Stillbirth
		Neonatal adiposity
**Long-term complications**	Impaired glucose metabolism in 50% of GDM cases	Obesity
	Type 2 DM- 20 times higher risk	Hyperinsulinemia
	Metabolic syndrome	Early onset cardiovascular disorders
	Cardiovascular disorders	High blood pressure
	Chronic inflammation	Attention-deficit hyperactivity disorder and autism spectrum disorders
	Chronic kidney disease	

**Table 4:** Proposed new strategies of screening and postpartum follow-up during the COVID-19 pandemic.

	RCOG	Canada	Australian
**Early pregnancy screening**	High risk women HbA1c or RPG	High risk women of overt diabetes HbA1c or FPG	High risk women HbA1c and random blood glucose
**24–28 weeks screening**	HbA1c and FPG or RPG	HbA1c and RPG	FBG OGTT if FBG> 4.7–5 mmol/L
**Postpartum follow-up**	HbA1c screening at 3–6 months	OGTT delayed until safe	OGTT delayed 6 months postpartum orHbA1c 3–6 months
